# A Simple Denoising Algorithm for Real-World Noisy Camera Images

**DOI:** 10.3390/jimaging9090185

**Published:** 2023-09-18

**Authors:** Manfred Hartbauer

**Affiliations:** Institute of Biology, University Graz, 8010 Graz, Austria; manfred.hartbauer@uni-graz.at

**Keywords:** night vision, real-world camera pictures, noise reduction, multi-core denoising, image enhancement, image processing, local means calculation

## Abstract

The noise statistics of real-world camera images are challenging for any denoising algorithm. Here, I describe a modified version of a bionic algorithm that improves the quality of real-word noisy camera images from a publicly available image dataset. In the first step, an adaptive local averaging filter was executed for each pixel to remove moderate sensor noise while preserving fine image details and object contours. In the second step, image sharpness was enhanced by means of an unsharp mask filter to generate output images that are close to ground-truth images (multiple averages of static camera images). The performance of this denoising algorithm was compared with five popular denoising methods: bm3d, wavelet, non-local means (NL-means), total variation (TV) denoising and bilateral filter. Results show that the two-step filter had a performance that was similar to NL-means and TV filtering. Bm3d had the best denoising performance but sometimes led to blurry images. This novel two-step filter only depends on a single parameter that can be obtained from global image statistics. To reduce computation time, denoising was restricted to the Y channel of YUV-transformed images and four image segments were simultaneously processed in parallel on a multi-core processor.

## 1. Introduction

The astonishing visual abilities of some insect species observable under extremely dim light conditions have attracted the attention of researchers for many years [1,2,3]. Nocturnal insects need to cope with the degradation of visual information arising from shot noise and transducer noise. Filtering in the spatial and temporal domains has been realized in denoising algorithms developed for removing noise from movies that were recorded under dim light conditions (e.g., [4,5]). The elimination of noise from static images is an even more difficult task because the temporal domain is not available for filtering. Currently, it is still challenging to remove noise from real-world camera images while avoiding artifacts and preserving object contours and image sharpness. The problem is that the noise statistics of camera images are very different from the Gaussian noise or salt-and-pepper noise often added to images to demonstrate the performance of denoising methods. The bionic night vision algorithm proposed by Hartbauer [6] was originally developed to remove noise from dim light images, but needs to be modified to become applicable to real-world noisy images exhibiting lower noise levels in three color channels. Here, I describe the modified version of this bionic spatial-domain-denoising algorithm and applied it to a real-world image dataset. 

Images taken with a CCD or CMOS camera under various light conditions often suffer from imperfections and sensor noise. In recent decades, the statistical property of real-world noise has been studied for CCD and CMOS image sensors [7,8,9,10]. Real-world noise has five major sources, including photon shot noise, fixed pattern noise, dark current, readout noise, and quantization noise (for further detail, see [11]). Therefore, the denoising of real-world images is still a challenging problem [10] and image databases containing noisy and noise-reduced camera images of the same scenes are needed. Xu et al., 2018 [11], computed the mean image from static scenes to obtain the “ground truth” image for real-world noisy camera images. Sampling the same pixel many times and computing the average value (e.g., 500 times) will approximate the truth pixel value and significantly remove image noise. The resulting image dataset was made available to the public (https://github.com/csjunxu/PolyU-Real-World-Noisy-Images-Dataset, accessed on 1 March 2023) and contains 40 different scenes captured using five cameras from the three leading camera manufactures: Canon EOS (5D Mark II, 80D, 600D); Nikon (D800); and Sony (A7 II). This image dataset consists of 100 images and was used in this study to test the performance of a modified version of the bionic night vision algorithm described by [6].

Typically, noise reduction can be achieved by applying linear and non-linear filters (for a review of methods, see [12,13]). Linear smoothing, or median filtering, can reduce noise, but at the same time smooth out edges, resulting in a blurred image. An alternative and improved denoising method is total variation minimization (TV) denoising, which has been described by [14]. The objective is the minimization of the total variation within an image, a concept that can be approximately characterized as the integral of the image gradient’s norm. Non-local means (NL-means) filtering represents an influential denoising filter technique that concurrently preserves image acuity and object contour fidelity [15]. Furthermore, bilateral filtering constitutes a robust non-linear denoising algorithm rooted in the consideration of spatial proximities among neighboring pixels alongside their radiometric congruence [16]. While bilateral filtering offers computational expediency, it poses challenges in the intricate calibration of its filter parameters [17], and it is recognized that this algorithm may yield artifacts such as staircase effects and inverse contours. Alternatively, image denoising can be accomplished through Fourier transformation of the original image, wherein Fourier-transformed images undergo filtration and subsequent inverse transformation, thereby mitigating noise and averting undesirable blurring phenomena (e.g., [18,19]). Frequency-domain methods are hindered by their propensity to introduce multiple undesirable artifacts and their inability to uniformly enhance all image components. In contrast, wavelet-domain hidden Markov models have exhibited intriguing outcomes in the context of image denoising, particularly when employed on diagnostic images [20,21,22]. In recent times, deep learning artificial neural networks (ANNs) have been employed for image denoising [23,24]. However, when contrasted with more straightforward denoising algorithms, the outcomes generated by ANN networks exhibit reduced predictability.

Recently, powerful algorithms have been developed for the denoising of real-world camera images to overcome the problem of different noise levels in the three color channels of color images [25] and the fact that noise is signal-dependent and has different levels in different local patches [11]. In the latter study, the authors proposed an algorithm that is based on the trilateral weighted sparse coding (TWSC) scheme of real-world color images. In contrast to denoising all color channels of RGB images with different levels of noise, denoising was only applied to the Y channel of YUV-transformed images in this study and a single hard threshold was used for an adaptive local averaging procedure [6] to enhance the quality of real-world noisy images with complex noise statistics. This simple algorithm was executed in parallel on a multi-core processor and the results were compared with four common denoising algorithms.

## 2. Bionic Method of Image Denoising

### 2.1. Method Overview

The presented image denoising approach enhances the quality of real-world photographs captured with cameras through the combination of two consecutive image processing stages. The initial stage involves pixel-level denoising, emulating the spatial information integration observed in nocturnal insects [1,2] and the second stage enhances image sharpness. The necessity for the second stage arises due to the advantageous improvement in signal-to-noise ratio achieved by aggregating visual information from a wide field of view, albeit at the potential cost of degrading image sharpness. To mitigate potential blurriness, it is necessary to adapt the extent of spatial summation, with smaller summation applied in regions of high contrast and larger summation in regions of greater image uniformity. This adaptive local averaging (ALA) represents the primary processing step in the algorithm, preserving object contours to a significant extent while introducing a slight reduction in image sharpness [6]. ALA operates under the assumption that luminance values exhibit higher variability near object contours compared to homogeneous image areas. Consequently, areas allowing local luminance value averaging are smaller in proximity to object contours and larger in regions with higher image uniformity. ALA is executed at the pixel level, employing a stringent threshold to assess the local gray value variability and identify the size of a quadratic region within which gray value variability remains below a predefined threshold, derived from global image statistics. Upon surpassing this threshold, the algorithm calculates the average luminance value for pixels within this region and stores it at the central pixel. Subsequently, in the second processing stage, an automatic procedure for enhancing image sharpness is applied using unsharp masking. The image-enhancement algorithm described here was programmed in Python (Version 3.8.3) and allows the execution of commands at the level of pixels using the openCV and PIL image libraries. 

### 2.2. Import Pictures

Real noisy images were imported into Python using the command cv2.imread() from the openCV image library. RGB images were converted into the YUV color scheme to isolate the brightness channel (Y) for image denoising. After denoising the Y channel, it was added to the original UV channels and the denoised image was converted back into the RGB color scheme.

### 2.3. Image Statistics

The ALA denoising algorithm only depends on a single parameter that defines a variability threshold for local averaging. This threshold (*Th*) parameter was derived from the global image statistics of the Y channel of the YUV color scheme after employing Equation (1). In this equation, the median gray value (µ) of an image was divided by 60 and sigma (σ) was used to estimate the noise from the absolute of the Laplacian transformed Y channel. The Laplacian of an image is the 2-D isotropic measure of the 2nd spatial derivative of an image and highlights regions of rapid intensity change.

(1) $$Th=\left(\sigma \times 2\right)+\frac{µ}{60}$$

#### 2.3.1. Image Processing Step 1: Adaptive Local Averaging (ALA)

Image noise was widely removed by means of a patented “adaptive local averaging” ALA procedure (PCT/EP2017/083061; international patent: WO 2018/122008) that was executed for every luminance value of the Y channel. This denoising method only depends on a single parameter (*Th*) and can be run in parallel on a multi-core processor. This denoising method evaluates the variability in the pixel brightness values in expanding quadratic patches of the image until reaching the pre-defined variability threshold *Th*. Then, the gray value of the focal pixel is defined as the average brightness of pixels belonging to this region. The minimum length of the expanding patch was 2 and its maximum was restricted to 40 pixels. 

ALA was executed in parallel using all four processors of a Dell™ computer (Łódź, Poland) equipped with an Intel^®^ Core™ i7-9700 CPU (Intel, Santa Clara, CA, USA). For this purpose, the Y channel was divided into four segments of the same size. In order to process the four image segments in parallel on the CPU, the multiprocessing. Pool as well as the pool.starmap functions were used.

#### 2.3.2. Image Processing Step 2: Image Sharpening

After execution of ALA, image sharpness was enhanced by means of an unsharp mask filter to obtain output images that are close to the ground-truth images obtained via extensive camera image averaging (see https://github.com/csjunxu/PolyU-Real-World-Noisy-Images-Dataset, accessed on 1 March 2023). For this purpose, a Gaussian blur filter operating with a radius of five pixels was applied to each color channel obtained after splitting the image into separate RGB channels. Then, each color channel was mixed with its blurred counterpart by executing Equation (2) (*b* = blue channel, *g* = green channel, *r* = red channel).

(2) $$b,g,r_{sharp}=\left(b,g,r\times 1.6\right)-(b,g,r_{sharp}\times 0.6)$$

Finally, the sharpened color channels were merged into an RGB image.

### 2.4. Common Noise Filters Applied to the Image Dataset

For the comparison of the denoising performance of the ALA filter with other commonly used denoising algorithms, all images from the real-world image dataset were denoised with bm3d (https://inria.hal.science/inria-00369582/document, accessed on 3 August 2023), bilateral (bilateral; ref. [16]), non-local means (NL-means; ref. [15]), total variation (TV filter; ref. [26]) and wavelet transform filters (wavelet; ref. [18]). These filters are included in the skimage.restoration Python library. The following command was used for the bilateral noise filtering of color images: denoise_bilateral(image, sigma_color = 2.0, sigma_spatial = 2.0, mode = ‘edge’, multichannel = True). TV filtering of color images uses a method that was described by [26] and was executed with the following command: denoise_tv_chambolle(image, weight = 0.01, multichannel = True). The wavelet filter was executed for color images with this python command: denoise_wavelet(image, sigma = sigma1, wavelet = ‘db2’, mode = ‘soft’, method = ‘BayesShrink’, multichannel = True, rescale_sigma = True). It also applies BayesShrink, which is an adaptive thresholding method that computes separate thresholds for each wavelet sub-band [18]. However, estimating the sigma1 parameter resulted in weak image denoising. Therefore, sigma1 was defined as 10 percent of the standard deviation of the image to improve denoising results. In contrast to these filters, the NL-means filter (for documentation, see non-local means denoising for preserving textures—skimage v0.20.0 docs (scikit-image.org, accessed on 3 August 2023)) was applied only to the Y channel of YUV-transformed images with this command: cv2.fastNlMeansDenoising(Y, destination = None, h = 5, template_window_size = 4, search_window_size = 4). The bm3d filter was executed on the Y channel of YUV-transformed images with the python command bm3d.bm3d(Y, sigma_psd = 7). The parameters of all filters were manually optimized for the real-world image dataset used in this study. 

### 2.5. Evaluation of Denoising Performance 

The performance of all denoising algorithms was evaluated by calculating the peak signal-to-noise ratio (PSNR) between the original (noisy) image and the denoised image (method described by [27]). All images were transformed into gray value images before calculating PSNR values using the arithmetic mean of the gray values. The resulting value is given in dB and indirectly quantifies the difference between the noisy input image and the output image. PSNR is the ratio of the maximum possible pixel value in the image relative to the mean squared error between the original and the denoised image. Equation (3) was used to calculate the PSNR values of the input and output images after transformation of the color images to gray value images. In this equation, µ denotes the arithmetic mean of the squared difference between two images.

(3) $$PSNR=20\times log\left[\frac{255}{\sqrt{µ\left[{\left(image1-image2\right)}^{2}\right]}}\right]$$

A high PSNR indicates low noise and high quality, whereas a low PSNR indicates high noise and low quality. However, high PSNR values also indicate a high similarity between two images, because the PSNR value is infinite for identical images. This may lead to a problem because high PSNR values also indicate a low denoising performance if the output image is very similar compared to the noisy input image. Therefore, visual inspection of the denoising performance is essential and PSNR values solely computed on output images were additionally calculated using Equation (4).

(4) $$PSNR=20\times log\left(\frac{255}{µ}\right)$$

In Equation (4), µ denotes the arithmetic mean of gray-value-transformed images. According to Equation (4), PSNR values of brighter images are lower. Therefore, care was taken to preserve the brightness of images after denoising. This was checked by comparing the average brightness before and after denoising.

## 3. Image Denoising Results

**Figure 1:**
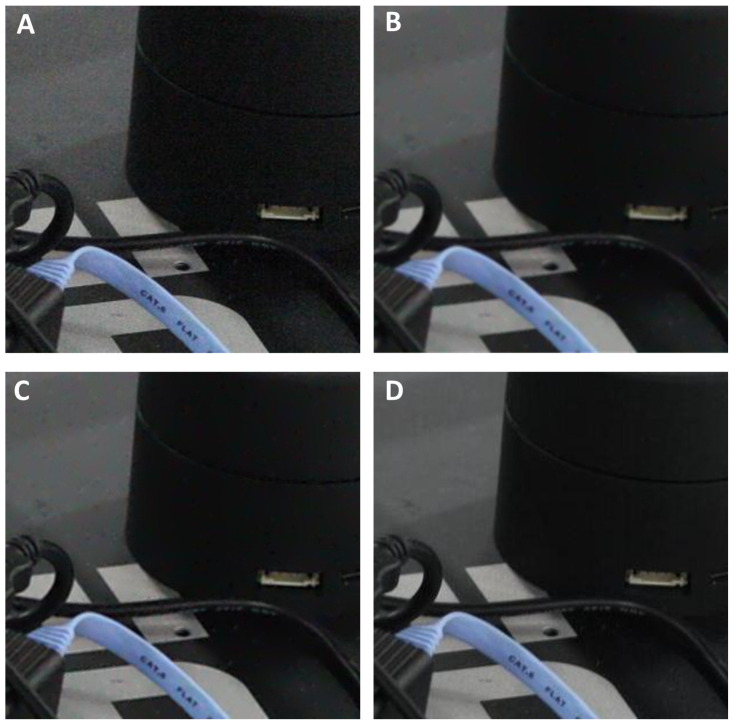
Example of the two-step image enhancement: (**A**) A segment of the real-world noisy image ‘Canon5D2_5_160_6400_reciever_8_real’. (**B**) ALA-filtered image shown in (**A**). (**C**) Unsharp mask-filtered image shown in (**B**). (**D**) Ground-truth image obtained via multiple averaging of this static scene. Note the high similarity between (**C**,**D**).

**Figure 2:**
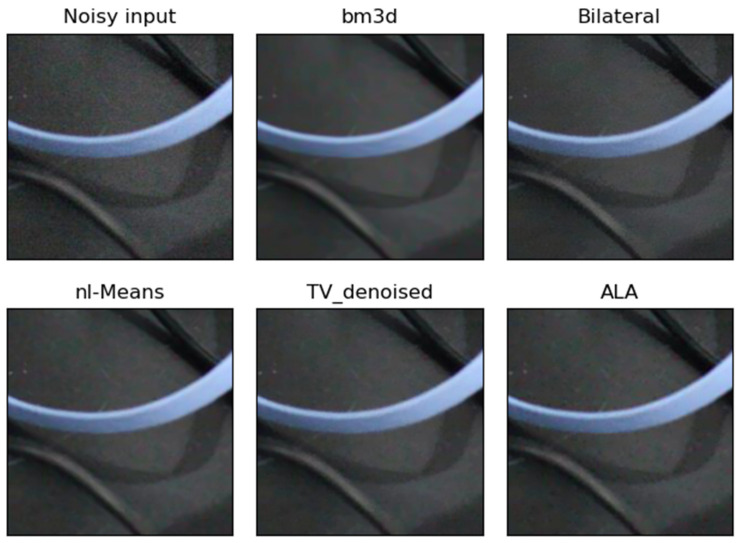
Example of the performance of different noise filters. The dark real-world noisy image “Canon5D2_5_160_6400_reciever_4_real” was taken with a Canon camera and a segment of this image is shown as “Noisy input”. The output of the ALA filter followed by the unsharp mask filter (ALA) is similar to the output of NL-means and TV denoising. The bilateral filter was imperfect in dark image regions, whereas bm3d filtering removed noise effectively.

**Figure 3:**
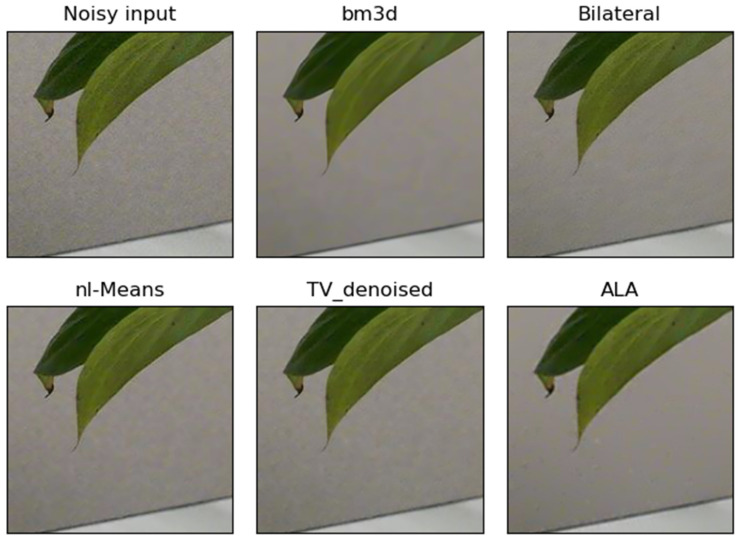
Example of the performance of different noise filters. The bright real-world noisy image “Sony_4-5_125_3200_plant_13_real” was taken with a Sony camera and a segment of this image is shown as “Noisy input”. The output of the ALA filter followed by the unsharp mask filter (ALA) is similar to the output of bm3d, NL-means and TV denoising. In this example, bilateral filtering did not remove noise effectively.

**Figure 4:**
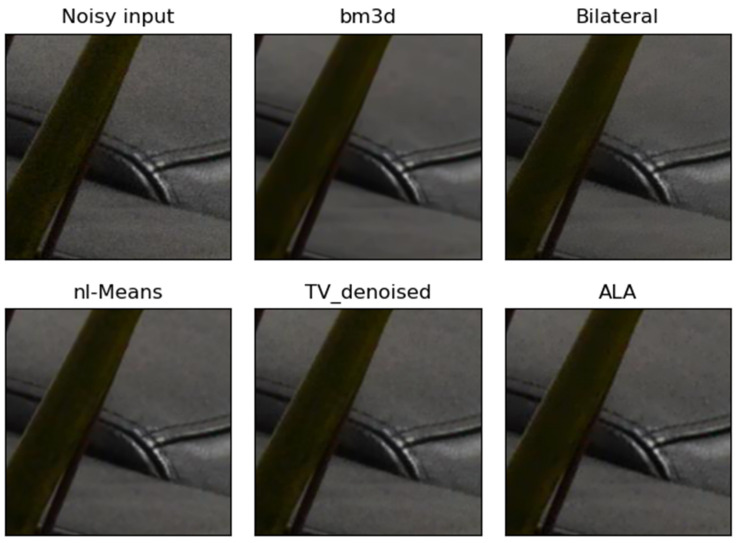
Example of the performance of different noise filters. The dark real-world noisy image “Ni-konD800_10_100_6400_planandsofa_7_real” was taken with a Nikon camera and a segment of this image is shown as “Noisy input”. The output of the ALA filter followed by the unsharp mask filter (ALA) is similar to the output of NL-means and TV denoising. In this example, bilateral filtering left some noise in dark image regions. Bm3d removed noise effectively, but the output appears slightly blurry.

**Figure 5:**
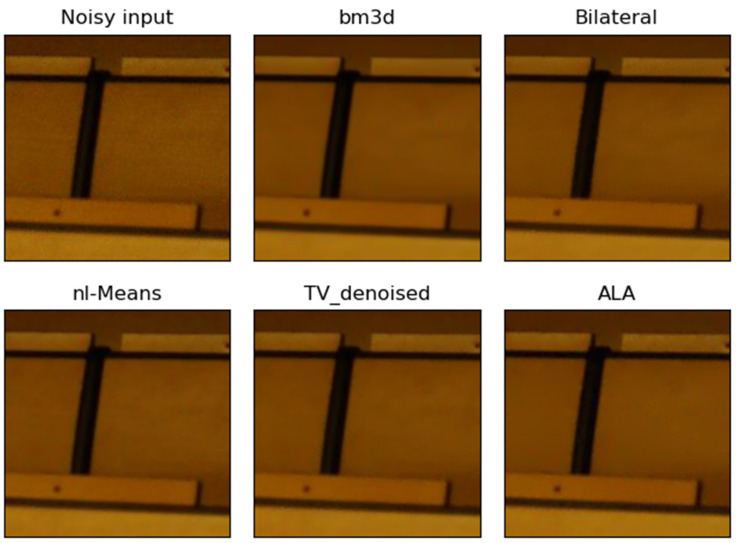
Example of the performance of different noise filters. The bright real-world noisy image “Sony_3-5_200_1600_classroom_13_real” was taken with a Sony camera and a segment of this image is shown as “Noisy input”. The output of the ALA filter followed by the unsharp mask filter (ALA) is similar to the output of the other noise filters in this example. All filters removed sensor noise effectively.

Adaptive local averaging (ALA) effectively removed sensor noise from the real-world images that were taken in various settings using five different camera models. However, the sharpness of ALA-filtered images was slightly reduced and it was necessary to enhance image sharpness in a second filter step (Figure 1). A visual image comparison shows that the performance of this two-step filter is comparable to the common image denoising filters NL-means and TV filter (for examples, see Figure 2, Figure 3, Figure 4 and Figure 5). Bm3d filtering performed the best regarding its denoising performance and in many cases preserved image sharpness. The bilateral filtering left some noise behind in dark image regions (Figure 2 and Figure 3) and the wavelet filter generated disturbing artifacts at object contours in the form of staircase effects. Therefore, the results for the wavelet filter are not shown in Figure 2, Figure 3, Figure 4 and Figure 5. The staircase artifact was completely absent in the output of the two-step image processing filter described in this study. 

ALA filtering of the whole real-world image dataset resulted in an average PSNR value of 38.0 ± 3.22 dB (Equation (3)) and additional image sharpening increased this PSNR value to 39.0 ± 2.75 dB. The average PSNR values calculated using Equation (3) were significantly higher for the five common denoising filters compared to the two-step filter described here (*p* < 0.01, N = 100, Mann–Whitney U test, see Table 1). Calculating PSNR values from the ground-truth images and denoised images (using Equation (3)) resulted in rather similar average PSNR values for all filters with slightly higher values for the bm3d, NL-means and TV filter compared to the two-step filter (see Table 1). In order to estimate the image denoising performance without comparing input and output images, PSNR values were also computed using Equation (4). The PSNR values of the output of the five common image denoising algorithms were very similar, but the application of two-step image denoising resulted in a slightly higher average PSNR value of 9.25 dB (see Table 1). However, this small difference between the PSNR values of filter variants is not significant (*p* > 0.05, N = 100, Mann–Whitney U test). The brightness of the output images was not affected by any filter applied in this study and was 96 for the whole image dataset for gray-value-transformed images. The mean SSIM index was very similar for all filter variants tested in this study (Table 1). Interestingly, SSIM was highest for the bilateral filter, which was less effective in removing sensor noise from images compared to the bm3d filtering, which showed better denoising performance. In contrast, when the ground-truth image was compared with filter output, mean SSIM was highest for the bm3d filter (see Table 1). 

Dividing images into four segments of equal size increased the processing speed, but at the same time resulted in line artifacts after application of the ALA filter. Therefore, an overlap of 20 pixels (equal to the maximum radius of the ALA filter) between adjacent image segments was necessary to enable parallel processing without generating image artifacts in the form of horizontal and vertical lines. The average processing speed of the two-step image denoising filter in a Python script environment (Pycharm version 2023.1 in Anaconda Python interpreter) was 36.6 ± 18.2 s for input images with a dimension of 512 × 512 pixels (cropped image dataset), which is about three times faster compared to single-core computing. A possible C-code compilation of the ALA filter function will further increase the processing speed.

## 4. Discussion

The filtering results show that the combination of “adaptive local averaging” (ALA) and image sharpening leads to high-quality output images when real-world camera images with complex noise statistics are taken as input (see Figure 2, Figure 3, Figure 4 and Figure 5). Visual inspection of the image denoising of this two-step filter shows that this special kind of local means filter is comparable to the performance of NL-means and TV filters. Bm3d filtering was more effective in removing sensor noise from images compared to the two-step filter, but some output images appeared slightly blurry (for example, Figure 4). In contrast, the bilateral filter was often less effective in removing noise from dark image regions and the wavelet filter generated artifacts in the form of staircase effects appearing next to object contours (data not shown). The ALA filter was originally inspired by the neuronal summation of adjacent photoreceptor cells of nocturnal insects, such as *Megalopta genalis*, where spatial integration of image information in lamina neurons leads to denoising and enables night vision [2]. The drawback of any local averaging filter is that output images are often blurry. Therefore, it was necessary to enhance image sharpness by applying an unsharp mask with a fixed radius of five pixels to obtain an image quality that was comparable to ground-truth images (see Figure 1).

PSNR values are often computed to compare the performance of different denoising filters. The similar PSNR values of all noise filters applied in this study (see Table 1) demonstrate a rather high performance of the two-step filter. The significantly lower average PSNR value of this filter, obtained by comparing the noisy input with the filtered output, is a consequence of the high number of tested images (N = 100). Using Equation (3) for PSNR calculation can be problematic because even weak denoising filters yield high PSNR values when the output image is similar to the noisy input (low denoising performance). This is also reflected in the high average SSIM value of the bilateral filter (see Table 1), which is unlikely the result of the denoising performance of this filter because bm3d filtering as well as all other filters removed sensor noise much more effectively (see Figure 2, Figure 3, Figure 4 and Figure 5). This problem with PSNR values also becomes obvious when comparing the SSIM values that were calculated for the ground-truth images and the denoised output (Table 1). In this case, the bm3d filter had the highest SSIM value, which indicates a high similarity between the filter output and ground truth images. Wavelet denoising had the lowest SSIM value, likely because of its staircase artifacts reducing image quality. The SSIM value obtained with the two-step filter described in this study was similar to the SSIM output of the bm3d, TV and NL-means filters when ground-truth images were compared with the corresponding filtered images. This result is surprising given the simplicity of the ALA filter, which only performs adaptive local averaging. Interestingly, using the same noisy image dataset as in this study, various elaborated denoising algorithms resulted in slightly lower PSNR values (maximum = 37.81 dB for the TWSC filter; ref. [11]). However, it is difficult to compare PSNR values between studies because several equations exist for calculating PSNR values and in this study the PSNR values were calculated after the gray value transformation of color images. To circumvent this problem, I calculated the PSNR values using Equation (4), which revealed a slightly higher average PSNR value for the two-step denoising compared to the five common denoising filters. 

In recent years, modern artificial neuronal network approaches have been developed for image denoising (e.g., [23,24]). Noise-free ground-truth images are essential for ANN training and the image dataset used in this study would offer this possibility, although the number of images is rather small for splitting the data into training and test datasets. In a follow-up study, the performance of the ALA filter will be compared with modern ANN-based noise filters using bigger image libraries such as the Smartphone Image Denoising Dataset (SIDD) consisting of 30,000 noisy images from 10 scenes.

A major benefit of the ALA filter is that it only depends on a single threshold parameter (*Th*) that can be derived from global image statistics (see Equation (1)). In contrast, several parameters need to be carefully adjusted for all other filters applied in this study. For example, the NL-means filter adjusts a smoothing parameter and four other parameters (tau, alpha, beta, gamma) that affect denoising performance and image sharpness. Using Equation (1), it was possible to calculate the *Th* for ALA denoising in a way that enhanced the quality of real-world noisy images exhibiting various levels of noise and different degrees of image brightness. To account for differences in image statistics, it was necessary to take the median image brightness and a kind of noise estimate into account for the calculation of the *Th* parameter for ALA denoising (see Equation (1)).

**Figure 6:**
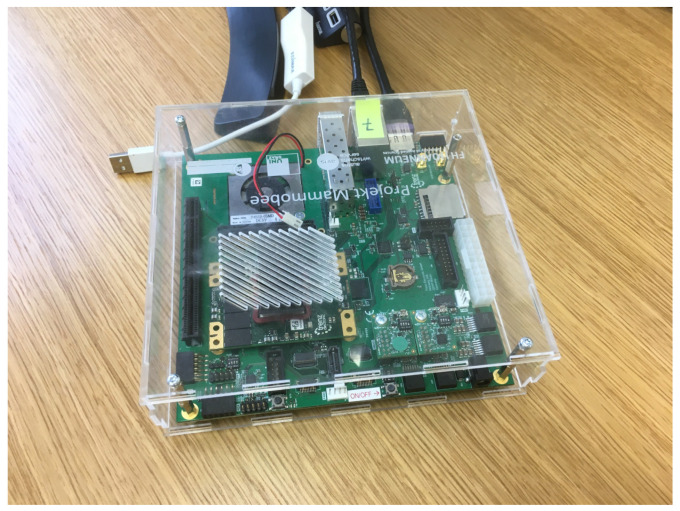
Trenz Electronic FPGA hardware enabling ALA parallel computation.

The ALA denoising described in this study is computationally demanding because the pixel-wise calculation of the diameter restricts local averaging to a small image region. To increase the processing speed, denoising was only performed on the brightness channel of YUV-transformed images. This saved 2/3 of the processing time compared to denoising all color channels of RGB images with different noise statistics that need to be taken into account for high-quality image denoising [28]. Another method of enhancing the processing speed was achieved by splitting input images into four equal segments for multi-core parallel processing. In this study, parallel processing improved the computation speed almost three times and depended on the number of processors that were simultaneously available. Nevertheless, none of these methods are sufficient to compute large images in a short period of time because the computation demand increases with image size in a non-linear way. For practical purposes, it will be necessary to compile the ALA algorithm as C-code to enhance the processing speed on standard computer hardware. Theoretically, it would also be possible to enhance the processing speed by running ALA on a multi-processor environment supported by modern computation clusters. Another solution constitutes fast computer graphics hardware (GPU) or FPGA (field-programmable gateway arrays) hardware. In the latter case, processing speed increases due to the parallel architecture of FPGA boards [27,29]. Simple algorithms, like the ALA filter, can be implemented in reconfigurable FPGA hardware [30], which is considered a practical way to obtain high computing performance (Xilinx Inc. System Generator for Digital Signal Processing; http://www.xilinx.com/tools/dsp.htm, accessed on 3 August 2023). Recently, image denoising based on the ALA filter was successfully implemented on a Trenz Electronic FPGA hardware platform for denoising high-resolution 16-Bit mammography images (the prototype is shown in Figure 6). This hardware enables parallel execution of ALA image denoising on many image segments at the same time. 

## 5. Conclusions

ALA image denoising relies on a single threshold parameter and operates in the spatial domain to successfully remove sensor noise from real-world camera images exhibiting various noise and brightness levels. In combination with an image-sharpening filter, this two-step image denoising method enhanced the quality of real-world images that were taken with different camera models in various light conditions. Compared to all other filters applied in this study, only one threshold parameter is required for effective noise removal using the ALA filter. The calculation of this threshold parameter only takes image brightness and an estimate of image noise into account. The denoising performance of this two-step filter is comparable to NL-means and TV denoising after visual inspection of filtered images. Compared to bm3d filtering, the two-step filter removed noise less effectively, but conserved image sharpness better. This led to similar SSIM values for both filters when ground-truth images were compared with denoised ones. In conclusion, the two-step filter exhibited good denoising performance on noisy real-world camera images, but did not perform better than more mathematically complex noise filters. ALA denoising was executed on a multi-core processor on the Y channel of YUV-transformed images, which reduced the computation time and simplified the noise estimation. This rather simple denoising algorithm also runs on FPGA hardware, which allows image processing to be executed in parallel at the level of adjacent pixels. 

## Figures and Tables

**Table 1:** Comparison of the performance of different noise filters.

	NL_Means	TV_filter	Bilateral	Wavelet	Bm3d	ALA+ Sharpened
Noisy vs. Denoised						
PSNR (dB), Mean	**43.2**	**43.4**	**43.1**	**40.3**	**40.5**	**39.0**
PSNR, STD	1.86	1.91	0.96	3.02	1.77	2.75
**SSIM**, Mean	**0.988**	**0.989**	**0.991**	**0.984**	**0.981**	**0.978**
SSIM, STD	0.0057	0.0049	0.0024	0.0074	0.0086	0.0093
Ground_truth vs. Denoised						
PSNR (dB), Mean	**39.1**	**39.2**	**38.3**	**37.9**	**39.7**	**38.0**
PSNR, STD	2.81	2.74	2.67	2.57	3.00	2.93
**SSIM**, Mean	**0.987**	**0.987**	**0.983**	**0.981**	**0.990**	**0.984**
SSIM, STD	0.0060	0.0057	0.0082	0.0078	0.0066	0.0088
Denoised image						
PSNR (dB), Mean	**9.19**	**9.19**	**9.19**	**9.19**	**9.24**	**9.25**
PSNR, STD	3.83	3.83	3.83	3.83	3.85	3.85

## Data Availability

Data sharing not applicable.
